# Behavioral driving through on line monitoring and activity-dependent stimulation in weakly electric fish

**DOI:** 10.1186/1471-2202-14-S1-P405

**Published:** 2013-07-08

**Authors:** Caroline G Forlim, Carlos Muñiz, Reynaldo D Pinto, Francisco B Rodríguez, Pablo Varona

**Affiliations:** 1 Departamento de Física Geral, Universidade de Sao Paulo, Sao Paulo, 05508-090, Brazil; 2 Escuela Politécnica Superior, Universidad Autonoma de Madrid, Madrid, 28049, Spain; 3 Instituto de Física de Sao Carlos, Universidade de Sao Paulo, Sao Carlos, 13560-970, Brazil

## 

On line monitoring and event-driven stimulation are promising techniques for neuroscience studies, especially in behavioral experiments [1]. Weakly electric fish have an electric organ and electroreceptors to generate and detect electric fields [2]. They use their electric pulses to 'see' their environment and also to communicate, changing their inter pulse intervals depending on the behavioral context. We conducted 2 behavioral monitoring experiments with *Gnathonemus petersii *where stimuli were triggered by (1) the fish position in the tank and (2) the fish's own electrical activity.

**Figure 1:**
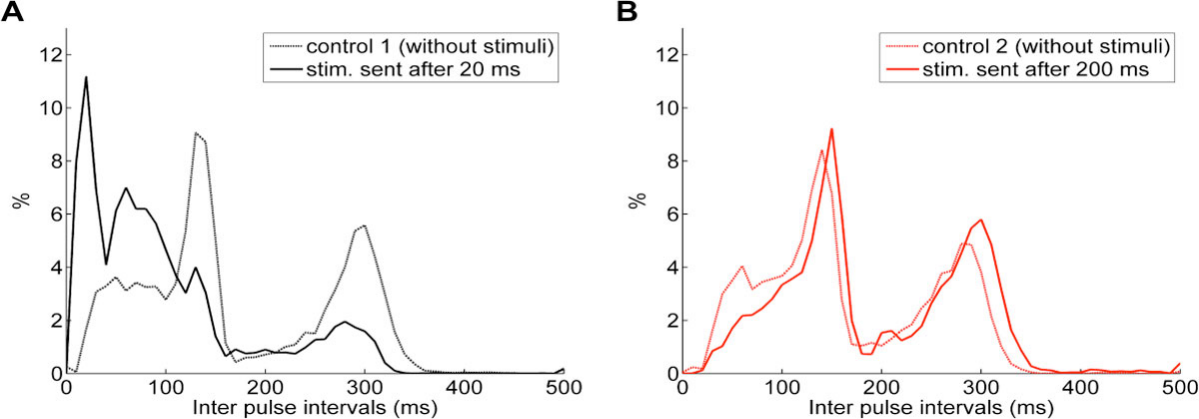
**IPI histograms when the fish was without stimulus for 30 min (dashed lines) and stimulated by a electric pulse sent 20 ms (A) and 200 ms (B) after detecting a pulse from the fish (black and red lines respectively)**. **A**. The fish increased its frequency (shorter IPIs) compared to the control with a new high peak in 11 ms and discharging less 130 ms and 300 ms IPIs. **B**. There was a decrease in the frequency of the fish (longer IPIs) under stimulation, the peaks changed from 140 ms to 150 ms and 290 ms to 300 ms.

(1) We built a virtual fence isolating the fish in a given area in the tank, using a camera and video-event driven stimulation. Fast on line tracking was achieved by subtracting consecutive frames. When the fish crossed the virtual fence, electric stimuli were delivered. We observed that artificial stimuli as high frequency signals were more efficient to create a virtual fence than pre-recorded *Gnathonemus petersii *waveforms from another fish.

(2) We monitored in real time the electrical activity of the fish and delivered electric pulses. Fish's electrical activity was acquired in real time from 5 dipoles by a DAQ board and the pulses from the fish were detected by a computer. Once a fish's pulse was detected, a 3 V pulse stimulus was delivered to the fish with a delay τ. Fish responded by shortening their inter pulse intervals (IPIs) for short τ values (Figure 1A) and discharging longer IPIS (Figure 1B) or not altering their IPIs for longer τ. We tested τ = 10 ms, 20 ms, 50 ms, 40 ms, 70 ms and 100 ms and we obtained similar results as shown in Figure 1A and τ = 160 ms, 200 ms, 280 ms, 400 ms with similar results as shown in Figure 1B.
